# Poverty and malaria in the Yunnan province, China

**DOI:** 10.1186/2049-9957-3-32

**Published:** 2014-09-01

**Authors:** Yan Bi, Shilu Tong

**Affiliations:** 1 School of Public Health and Social Work, Institute of Health and Biomedical Innovation, Queensland University of Technology, Victoria Park Road, Kelvin Grove, Brisbane, Australia

**Keywords:** Malaria, Poverty, Spatial and temporal distribution, International border area, Malaria elimination

## Abstract

Poverty and malaria appear to have an intertwined link. This paper aims to define the relationship between poverty and malaria in Yunnan, China, and to make recommendations for future research in this important area. Data on malaria prevalence and the population’s income in each county between 2005 and 2010 were obtained from the Yunnan Center for Disease Control and Prevention and the Yunnan Bureau of Statistics, respectively. Geographic mapping shows an apparent spatial convergence of poverty and the incidence of malaria at a county level, and suggests that poverty may be one of the drivers of malaria transmission in Yunnan. Future research should focus on: 1. measuring and quantifying the relationship between poverty and the malaria burden at the individual, community, county and regional level in Yunnan; and 2. developing the GIS-based spatial decision support system (SDSS) framework in malaria endemic areas, particularly along the border areas in Yunnan.

## Multilingual abstracts

Please see Additional file 1 for translations of the abstract into the six official working languages of the United Nations.

## Background

Malaria is one of the most serious vector-borne diseases (VBDs) and remains a leading cause of morbidity and mortality in the world. In 2011, epidemics of malaria still occurred in 104 countries, affecting health and wealth with approximately 216 million cases and about 665,000 deaths worldwide. International funding for malaria control increased sharply from less than US$ 100 million in 2000 to US$ 1.84 billion in 2012 [1]. The association between malaria and poverty is often inextricably intertwined: malaria may result in poverty and, in turn, poverty may aggravate malaria transmission [2]. There are an estimated 35 million disability-adjusted life years (DALYs) attributable to malaria each year [3]. Malaria results in a heavy burden of disease and is a threat to global health, as well as to economic growth and development [2,3].

In China, the malaria elimination programme (MEP) was launched in 2010. The ultimate goal of the MEP is to interrupt the local transmission of malaria in all areas by 2015, excluding the border areas of the Yunnan province as these areas will remain endemic after 2015 due to the impact of imported cases from neighbouring countries. The number of malaria cases in Yunnan was the highest in China and accounted for 34% of the total cases in the country in 2011 [4]. Yunnan suffers from poverty due to its low Gross Domestic Product (GDP) level, which was ranked the 23^rd^ among the 30 provinces in China in 2010 [5].

This paper aims to define the relationship between poverty and malaria in Yunnan, China, and to make recommendations for future research in this important area. Data on malaria prevalence and the population’s income in each county between 2005 and 2010 were obtained from the Yunnan Center for Disease Control and Prevention and the Yunnan Bureau of Statistics, respectively. ArcGIS 10.0 (ESRI Inc., Redlands, CA) was used for geocoding and mapping.

## Main text

### Temporal distribution of poverty and malaria transmission in Yunnan

**Figure 1:**
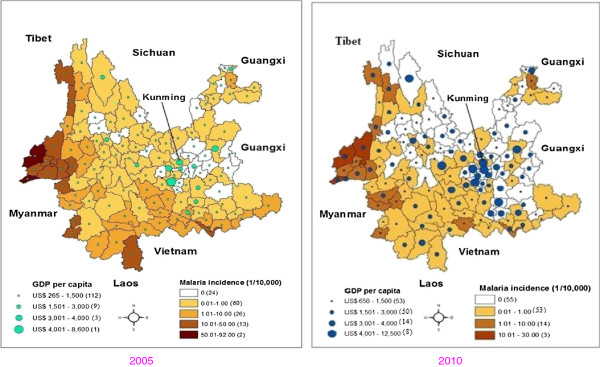
Temporal distribution of per capita GDP and malaria incidence in the Yunnan province, China.

Figure 1 shows the relationship between the temporal changes of annual GDP per capita and the annual malaria incidence in the Yunnan province. Notably, income levels increased during the study period. Counties with the lowest income level (under US $1,500 per capita annually) sharply decreased from 112 counties in 2005 to 53 counties in 2010.The number of counties with an annual income of US $1,501 to US $3,000 per capita increased from nine in 2005 to 50 in 2010. The number of counties with an annual income of US $3,001 to US $4,000 per capita increased sharply from three in 2005 to 14 in 2010, and the number of counties with the highest income (i.e., above US $4,000 per capita annually) increased remarkably from one in 2005 to eight in 2010.

Interestingly, the incidence of malaria declined in the Yunnan province between 2005 and 2010. Consistent with the changes in poverty, the number of counties with malaria incidence above 10.0/10,000 decreased from 15 counties in 2005 to only three in 2010, and the number of counties with malaria incidence between 1.0 and 10.0/10,000 was also reduced significantly, from 26 in 2005 to 14 in 2010. The number of counties with low levels of malaria incidence (i.e., 0.01–1.0/10,000) decreased slightly from 63 counties in 2005 to 53 counties in 2010, while the number of counties with no endemic malaria increased strikingly from 24 counties in 2005 to 55 counties in 2010.

### Spatial distribution of poverty and malaria transmission in Yunnan

**Figure 2:**
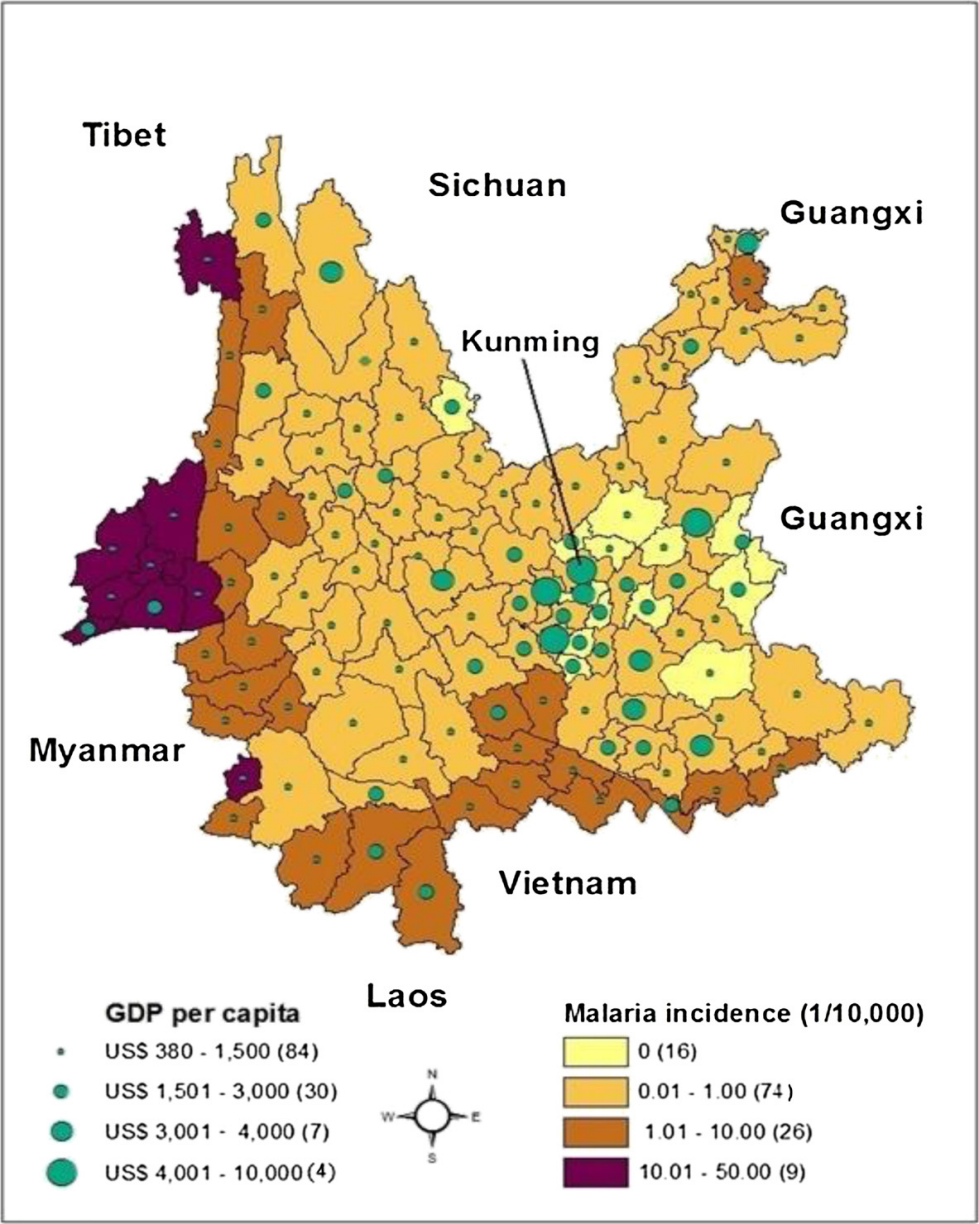
**Spatial distribution of malaria incidence and per capita GDP in the Yunnan province, China, 2005–2010.** (The spatial pattern of income is unevenly distributed, with average income levels significantly lower along the border and western areas. The spatial variation of malaria risk at a county level shows a disease burden that concentrates along the border area. The number in the parentheses denotes how many counties there are in each category. Kunming is the capital city of the Yunnan province and includes four main urban districts. Green pie: average annual GDP per capita, US$; Choropleth map: average annual malaria incidence, 1/10,000).

Between 2005 and 2010, the spatial pattern of the average annual GDP per capita at a county level in Yunnan (see Figure 2) shows an uneven distribution of economic statuses. From the total 128 counties in Yunnan, income levels were significantly lower along the border areas and in western Yunnan than in the other counties. The majority of counties (84) had a GDP per capita under US $1,500. All four counties with the highest annual income (i.e., US $4,001 to US $10,000 per capita) were around the capital city, Kunming.

Corresponding to the spatial pattern of poverty, there is a higher risk of malaria along the border areas as compared to the other areas in Yunnan. Among the 26 counties along the international border, nine counties had an incidence of malaria above 10.0/10,000, 15 counties between 1.0 and 10.0/10,000 and only two counties below 1.0/10,000. Among them, 21 counties have the lowest economic statuses (US $380 to US $1,500 per capita annually). The other five are also among the poorest (US $1,501 to US $3,000 per capita annually). A clear inverse relationship between income and malaria incidence was observed.

In Yunnan, there are some remote, forested and hilly regions which share a long border (about 4,060 km) with three malaria endemic countries: Myanmar, Laos and Vietnam. The province has a culture of ethnic diversity, with 25 ethnic minorities. Although the incidence of malaria was significantly reduced in Yunnan between 2005 and 2010, malaria transmission was still common on the border and in poorer areas (see Figure 2). The rural population along the border areas is among the poorest in China (average annual GDP per capita: < US$ 100) [6]. Yunnan has historically had stable endemicity of malaria and still has a higher level of local malaria transmission than any other province in China. In recent years, with the increase of the millions of cross-border migrants, malaria poses a significant threat to ethnic minorities with a low socioeconomic status in Yunnan [7]. Thus, there is a clear need to scale up the MEP in this province.

### Key challenges and research needs in malaria endemic areas

As no research on the burden of disease has been carried out for malaria in Yunnan, the specific burden of malaria and its health cost in the endemic areas and in the entire province remain largely unknown. Therefore, it is necessary to quantify the burden of malaria through further research. Historically, and even up to now, Yunnan has been suffering from a heavy burden of malaria transmission. For instance, in both 2005 and 2006, the number of malaria deaths in Yunnan accounted for more than 80% of the total deaths in China [8-10]. However, the estimation of malaria deaths should be conducted separately for *Plasmodium vivax* and *Plasmodium falciparum* as both types of parasites are prevalent in Yunnan. A measure of the burden of malaria can provide policymakers with useful evidence to support and evaluate the operation of the MEP in this province.

The results of this study show an apparent spatial convergence of poverty and the incidence of malaria at a county level, and suggest that poverty may be one of the drivers of malaria transmission in Yunnan. In addition to the local malaria transmission, Yunnan faces an increasing risk of imported malaria infections from neighbouring countries. There is a lack of active surveillance and no appropriate techniques to effectively evaluate the MEP at present [11]. In Yunnan, overseas-imported malaria infections have begun to dominate. In 2011, overseas-imported malaria infections accounted for more than 70% of all malaria cases in this province. An introduction of a GIS-based spatial decision support system (SDSS) is able to strengthen and validate the current surveillance-response system. The SDSS has been successfully applied in the Pacific Islands for malaria elimination programmes [12]. Under the SDSS framework, case detection (both passive and active) and rapid notification is an optimal solution to the deficiency of the current surveillance system. Furthermore, its monitoring and evaluation function is able to facilitate implementation of the MEP and evaluate malaria elimination activities, particularly in the resource-poor settings in Yunnan [13].

## Conclusion

There was an apparent link between poverty and malaria at a county level in Yunnan, and residents living along the border suffer more from poverty and malaria infections than those in the other regions of this province. There are two urgent research needs: 1. Measuring and quantifying the relationship between poverty and the malaria burden at the individual, community, county and regional level in Yunnan. This will provide the most important information that is needed to facilitate the on-going poverty reduction and malaria elimination activities and contribute to the protection and improvement of population health in this province. 2. Developing the GIS-based SDSS framework in malaria endemic areas, particularly along the border areas in Yunnan, which will support and assist in evaluating a causal relationship between poverty and malaria, and thus improve the current malaria surveillance-response system, particularly at a transition stage from malaria control to malaria elimination in Yunnan.

### Ethical approval

This study has obtained ethnical approval from both the Queensland University of Technology and the Yunnan Center for Disease Control and Prevention.

## Competing interests

The authors declare that they have no competing interests.

## Authors’ contributions

YB conceived the study, conducted the data collection and analysis, and drafted the manuscript. ST provided intellectual input and critically revised the manuscript. Both authors read and approved the final manuscript.

## Supplementary Material

Additional file 1 Multilingual abstracts in the six official working languages of the United Nations. Click here for file
