# Supplementary material for: Poverty and malaria in the Yunnan province, China
Source: Infect Dis Poverty. 2014 Sep 1;3:32. doi: 10.1186/2049-9957-3-32 (PMC4161269; doi:10.1186/2049-9957-3-32)
Supplement: Additional file 1 — Multilingual abstracts in the six official working languages of the United Nations. [file 2049-9957-3-32-S1.pdf]

Translation of the abstract into the six official working languages of the United Nations

الفقر والملاريا في مقاطعة يونان في الصين

يان بي وشي – لو تونغ

خلاصة

يبدو أن الفقر والملاريا متضافران. تهدف هذه الورقة إلى تحديد العلاقة الكامنة بين الفقر والملاريا في يونان في الصين، ولتقديم توصيات لبحوث مستقبلية في هذا المجال المتصف بالأهمية. وتوفرت تباعاً البيانات بشأن انتشار الملاريا ودخل السكان في كل مقاطعة للفترة المتراوحة ما بين عام 2005 و2010 من مركز يونان لمكافحة الأمراض والوقاية منها ومن مكتب يونان للاحصائيات. وتظهر الخريطة الجغرافية تقارباً مكانياً ظاهراً بين الفقر والإصابة بالملاريا على المستوى القطاعي، وتقترح أن الفقر قد يكون أحد عوامل انتقال عدوى الملاريا في يونان. لذا، يجدر بالبحوث المستقبلية أن تركز على: 1. قياس العلاقة بين الفقر وعبء الملاريا وتقديرها كمياً على المستوى الفردي والمجتمعي والقطاعي والإقليمي في يونان؛ 2. تعزيز إطار نظام دعم القرار المكاني (SDSS) المرتكز على نظام المعلومات الجغرافية في المناطق الموبوءة بالملاريا، وبشكل خاص على طول المناطق الحدودية ليونان.

Translated from English version into Arabic by Liliane Hatem, through

TRANSLATORS
WITHOUT BORDERS

中国云南疟疾与贫困的关系

Yan Bi and Shilu Tong

摘要

疟疾似乎常和贫困联系在一起。本文旨在探讨云南省疟疾和贫困的关联，并为今后的研究方向提出建议。我们收集了 2005-2010 年云南省各县的疟疾发病和人均收入的资料，地理信息图清晰表明云南省疟疾和贫困密切相关，提示贫困可能是云南省疟疾传播的主要决定因素之一。今后的研究可侧重在两个方面：一是多层次定量分析疟疾和贫困的关系；二是在云南省疟疾传播的主要地区发展和采用基于地理信息系统的空间决策支持系统，尤其是边境地区。

Translated from English version into Chinese by Shi-lu Tong, through

TRANSLATORS
WITHOUT BORDERS

Pauvreté et paludisme dans la province du Yunnan, Chine

Yan Bi et Shi-lu Tong

Résumé

La pauvreté et le paludisme semblent être intimement liés. Ce document vise à définir la relation entre pauvreté et paludisme dans le Yunnan, en Chine, et à formuler des recommandations pour la recherche future dans cet important domaine. Les données sur la prévalence du paludisme et du revenu de la population dans chaque district entre 2005 et 2010 ont été obtenues du Centre de prévention et de contrôle des maladies de la province du Yunnan et du Bureau des statistiques du Yunnan, respectivement. La cartographie géographique montre une convergence spatiale apparente de la pauvreté et de l'incidence du paludisme au niveau du district, et laisse à penser que la pauvreté pourrait être l'un des moteurs de la transmission du paludisme dans le Yunnan. Les recherches futures devraient se concentrer sur : 1. mesurer et de quantifier la relation entre la pauvreté et le fléau que constitue le paludisme sur le plan individuel, de la communauté, du district et de la région du Yunnan ; et 2. élaboration du cadre de système d'aide à la décision spatiale basé sur le SIG dans les zones endémiques de paludisme, en particulier le long des zones frontalières du Yunnan.

Translated from English version into French by Christophe Delaunay, through

TRANSLATORS
WITHOUT BORDERS

Бедность и малярия в провинции Юньнань, Китай

Ян Би и Ши-лу Тонг

Выдержка

Вероятно, существует зависимость между бедностью и малярией. Настоящий документ призван определить взаимосвязь между бедностью и малярией в провинции Юньнань (Китай) и дать рекомендации для будущих исследований в этой важной области. Данные о распространении малярии и доходах населения в каждом уезде в период с 2005 по 2010 год получены соответственно в Центре контроля и профилактики заболеваний провинции Юньнань и Бюро статистики провинции Юньнань. Картографирование отражает очевидное пространственное сближение бедных регионов и мест заболеваемости малярией на уровне уезда, позволяя предположить, что бедность может быть одной из причин распространения малярии в провинции Юньнань. Будущие исследования необходимо направить: 1. на измерение и количественную оценку взаимосвязи между бедностью и бременем малярии на уровне индивидуума, общины, уезда и региона в провинции Юньнань; а также 2. на разработку пространственной системы поддержки решений (ПСПР) на основе ГИС-технологий в зонах заболевания малярией, особенно вдоль приграничных областей провинции Юньнань.

Translated from English version into Russian by Maryia Vasiuchenka, through

TRANSLATORS
WITHOUT BORDERS

La pobreza y la malaria en la provincia de Yunnan, China

Yan Bi y Shi-lu Tong

Resumen

La pobreza y la malaria parecen estar relacionadas. Este documento tiene como objetivo definir la relación entre la malaria y la pobreza en Yunnan, China y formular recomendaciones para futuras investigaciones en esta importante área. Los datos concernientes a la prevalencia de la malaria y el salario de la población en cada municipio, entre 2005 y 2010, se obtuvieron del Centro Yunnan para el Control y la Prevención de Enfermedades y de la Oficina de Estadísticas de Yunnan, respectivamente. Un mapeo geográfico, a nivel de municipio, muestra una convergencia espacial aparente entre la pobreza y la incidencia de la malaria y sugiere que, la pobreza puede ser una de las causas de la transmisión de la malaria en Yunnan. La investigación futura debe centrarse en: 1. Medir y cuantificar la incidencia de la relación entre la pobreza y la malaria, tanto a nivel individual, de la comunidad, del municipio y de la región de Yunnan; 2. Desarrollar una infraestructura de sistemas de ayuda a la decisión espacial[1] (SDSS, por sus siglas en inglés), basado en un sistema de información geográfica[2] (GIS, por sus siglas en inglés) en las zonas endémicas de malaria, particularmente, a lo largo de las zonas fronterizas de Yunnan.

Translated from English version into Spanish by Andrea Pisera, through

TRANSLATORS
WITHOUT BORDERS

---

[1] Sistema de Ayuda a la Decisión Espacial (SADE)

[2] Sistema de Información Geográfica (SIG)
